# Magnetocardiography in the diagnosis of suspected ANOCA: a case series

**DOI:** 10.3389/fcvm.2026.1787320

**Published:** 2026-06-19

**Authors:** Xiaoyan Xue, Jiandong Zhang, Yuheng Zhou, Lei Cheng, Yihao Zhu, Jiaqi Liang, Kangqi Tian, Dong Xu, Shuting Xiang, Qian Li, Jun Xiao, Yu Ma, Xu Zhang, Min Xiang

**Affiliations:** 1 Key Laboratory of Ultra-Weak Magnetic Field Measurement Technology, Ministry of Education, School of Instrumentation and Optoelectronic Engineering, Beihang University, Beijing, China; 2 Hangzhou Innovation Institute, Beihang University, Hangzhou, China; 3 Shandong Key Laboratory: Magnetic Field-free Medicine & Functional Imaging, Jinan, China; 4 Research Institute of Shandong University: Magnetic Field-free Medicine & Functional Imaging, Jinan, China; 5 Senior Department of Urology, Chinese PLA General Hospital, Beijing, China; 6 State Key Laboratory of Traditional Chinese Medicine Syndrome/National Institute of Extremely-weak Magnetic Field Infrastructure, Hangzhou, China; 7 Department of Cardiovascular Medicine, Chongqing Emergency Medical Center, Chongqing University Central Hospital, Chongqing, China; 8 Department of Nuclear Medicine, Chongqing Emergency Medical Center, Chongqing University Central Hospital, Chongqing, China; 9 Hefei National Laboratory, Hefei, China

**Keywords:** ANOCA, case report, chest pain, MCG, SERF atomic magnetometer

## Abstract

Patients with angina pectoris with non-obstructive coronary artery disease (ANOCA) often present to cardiology outpatient clinic or emergency departments with manifestations mimicking acute coronary syndrome (ACS). As a working diagnosis defined by angina pectoris in the absence of obstructive coronary arteries, ANOCA requires a stepwise diagnostic process—one that incorporates coronary functional assessments and provocative tests. Magnetocardiography (MCG) is a diagnostic modality with the potential to distinguish mild myocardial ischemic in patients with suspected ANOCA from nonischemic cardiac presentations, thereby shortening the diagnostic timeline and optimizing the clinical workflow. Three patients with angina pectoris and non-obstructive coronary arteries on CCTA were referred for MCG evaluation. We evaluated ischemic features on MCG waveforms. Notably, even in cases where ischemia could not be identified via electrocardiography (ECG), the ischemic features on MCG waveforms remained highly prominent. This study describes the clinical and MCG features of patients with suspected ANOCA, explores MCG's utility for diagnosing coronary microvascular dysfunction (CMD), and informs future research. The application potential of MCG in cardiology—specifically for the evaluation of acute chest pain—has recently garnered significant attention. To our knowledge, prior investigations in the context of ANOCA or CMD have not focused on the systematic characterization of specific MCG waveform patterns. Our documentation of positive MCG findings in these patients presenting with suspected ACS but having non-obstructive arteries supports MCG's potential in detecting subtle ischemia and optimizing clinical workflow.

## Introduction

Acute chest pain is a common reason for emergency department (ED) visits (1). Notably, up to 30% of patients with acute coronary syndrome (ACS) who undergo coronary angiography are found to have no culprit lesion on invasive coronary angiography (CAG) or coronary computed tomography angiography (CCTA)—a clinical conundrum in daily practice (2, 3). Angina pectoris with non-obstructive coronary artery disease (ANOCA) represents a working diagnosis for this population, which necessitates a standardized, stepwise diagnostic approach aimed at characterizing the underlying coronary functional abnormality. According to contemporary definitions, ANOCA describes the clinical phenotype of angina in the absence of obstructive coronary artery disease. The term ischemia with non-obstructive coronary arteries (INOCA) is applied when objective evidence of myocardial ischemia is additionally confirmed. Coronary microvascular dysfunction (CMD) is one of the key endotypes that can underlie both ANOCA and INOCA. Therefore, identifying the specific endotype is a critical diagnostic goal. This functional assessment, which is crucial for confirming the diagnosis and distinguishing between endotypes (e.g., microvascular dysfunction vs. vasospasm), can be supported by findings from non-invasive tests such as exercise stress testing or myocardial scintigraphy (4). Current chest pain guidelines recommend non-invasive cardiac testing (5) for patients with chest pain and non-obstructive coronary arteries who fall into the intermediate-risk category for acute coronary syndrome. In contrast, invasive functional testing is infrequently performed in modern clinical practice, largely due to logistical challenges, concerns about complications, increased cost and lack of expertise (6). Alternatively, non-invasive imaging modalities—including echocardiography, cardiac magnetic resonance imaging (CMR), and single-photon emission computed tomography (SPECT)—are often used in the initial assessment of patients suspected of having ANOCA. However, each of these three common non-invasive tools for evaluating coronary circulation has inherent limitations: SPECT is associated with ionizing radiation exposure; pharmacological stress echocardiography carries inherent risks that limit its applicability in certain clinical scenarios; and CMR can only serve as an auxiliary tool to “rule out other myocardial lesions” (e.g., myocardial infarction and cardiomyopathy), rather than a primary method for directly identifying or quantifying microvascular dysfunction in the diagnostic work-up of suspected ANOCA. Given the limitation of existing assessment methods for patients with symptoms and findings suggestive of ANOCA, selecting an appropriate ischemia evaluation approach is crucial for the rational assessment of this condition.

**Figure 1:**
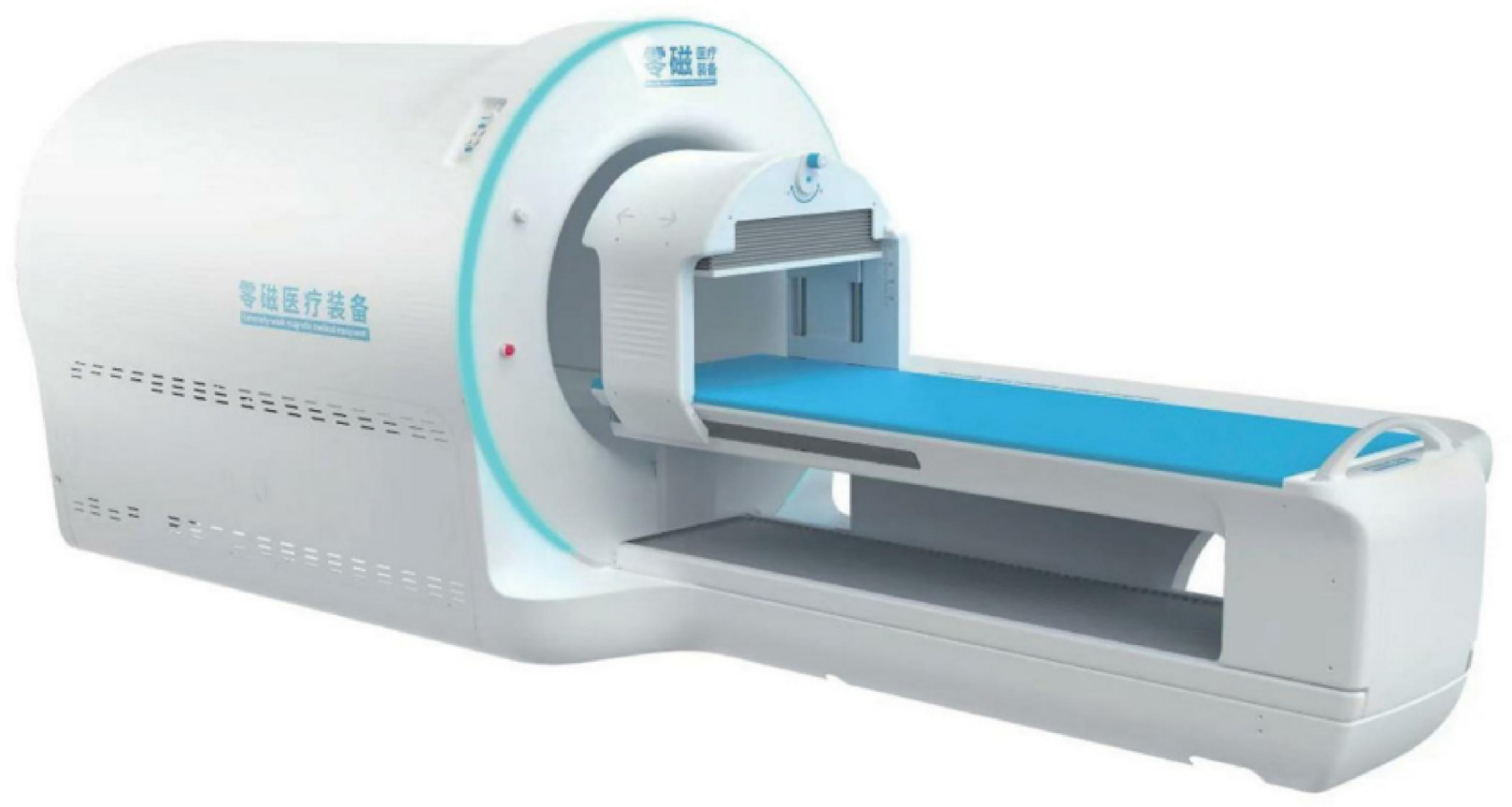
Image of the SERF-MCG magnetocardiograph system.

Magnetocardiography (MCG) is a rapid, non-invasive modality for mapping the weak magnetic field generated by the heart's intrinsic electrical activity. Although the application potential of MCG in cardiology—particularly for the evaluation of acute chest pain—has recently attracted substantial attention, prior investigations in the context of CMD or ANOCA have primarily focused on establishing abnormalities in two-dimensional isofield contour maps and pseudo-current-density maps. To our knowledge, no study has yet systematically characterized the specific MCG waveform patterns (e.g., ST-T morphology) associated with microvascular angina. Notably, MCG may offer the potential to optimize the diagnostic workflow for patients with this condition. To explore this potential, this case series investigates investigates the clinical application of MCG captured by the 36-channel spin exchange relaxation free magnetocardiography (SERF-MCG, Hangzhou Lingci Medical Equipment, LMCG-36A), as a rapid diagnostic tool in the cardiology outpatient clinic setting (Figure 1).

**Figure 2:**
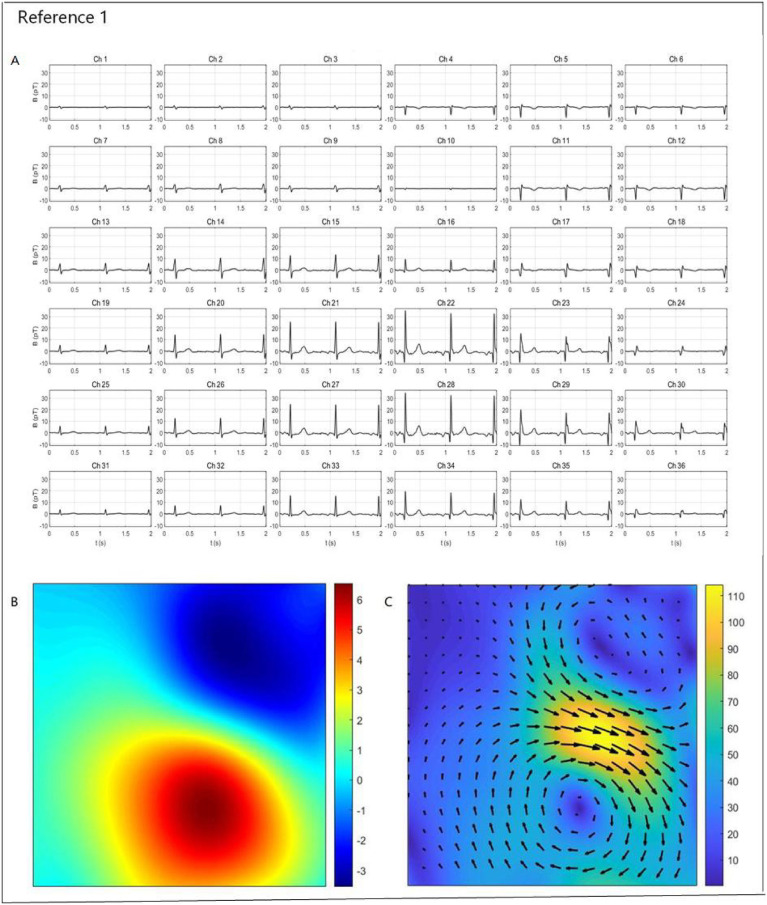
Representative MCG patterns from reference subjects and a patient with obstructive coronary artery disease. Reference 1 and 2 show normal MCG waveforms from two healthy volunteers: a 47-year-old male and a 50-year-old female, respectively. Both were recruited from the health examination center, had no cardiac history, and underwent MCG under the same standard recording conditions. Abnormal Features displays MCG patterns from a 48-year-old female patient with unstable angina and angiographically confirmed obstructive coronary artery disease, recruited from the cardiology outpatient clinic of the same hospital. The ischemic features, indicated by red arrows and boxes, correspond to characteristic waveform abnormalities associated with coronary ischemia. Both the magnetic-field map and the current-density map shown were acquired at the T-wave peak. Although the reference and patient data were acquired on MCG systems located in different departments, both systems were of the same model (LMCG-36A, Hangzhou Lingci). All waveforms were processed and normalized using identical software and analytical pipelines as detailed in the Methods, ensuring that the morphological features are comparable for illustrating distinct patterns between health and disease states. For all panels, the color scales of the pseudo-current-density maps have been optimized individually for visualization.

Similar to ECG waveforms, one-dimensional timeline MCG (TLMs) may serve as the mainstay for detecting coronary ischemia. Based on our previous analysis of TLM characteristics in patients with known obstructive coronary artery disease or ischemia confirmed by SPECT, we found that TLMs in ischemic patients exhibit flattened or downsloping ST segments and flattened or inverted T waves—changes that are absent in healthy individuals (Figure 2). Meanwhile, we performed a comparative analysis of two-dimensional magnetic field maps and current density maps between this group of patients with established ischemia (via TLMs) and healthy controls. We identified distinct morphological differences in TLMs between patients with symptoms and findings suggestive of ANOCA and healthy controls, and these differences were further corroborated by variations in two-dimensional magnetic field maps and current density maps. Collectively, these findings indicate that the aforementioned ischemic TLM characteristics can serve as easily identifiable indicators to distinguish patients with suspected myocardial ischemia (such as those with suspected ANOCA) from healthy individuals (Figure 2). Therefore, we used TLMs and two-dimensional MCG data from patients with established coronary ischemic disease and healthy individuals as references. Herein, we present MCG waveforms from 3 patients with angina and non-obstructive coronary arteries. We further evaluated the abnormal characteristics of these MCG waveforms as part of a clinical study conducted in the ED of a tertiary academic medical center. Our primary aim was to explore the concordance of MCG findings with suspected microvascular myocardial ischemia and assess its potential clinical value in the evaluation of patients with angina and non-obstructive coronary arteries.

**Figure:**
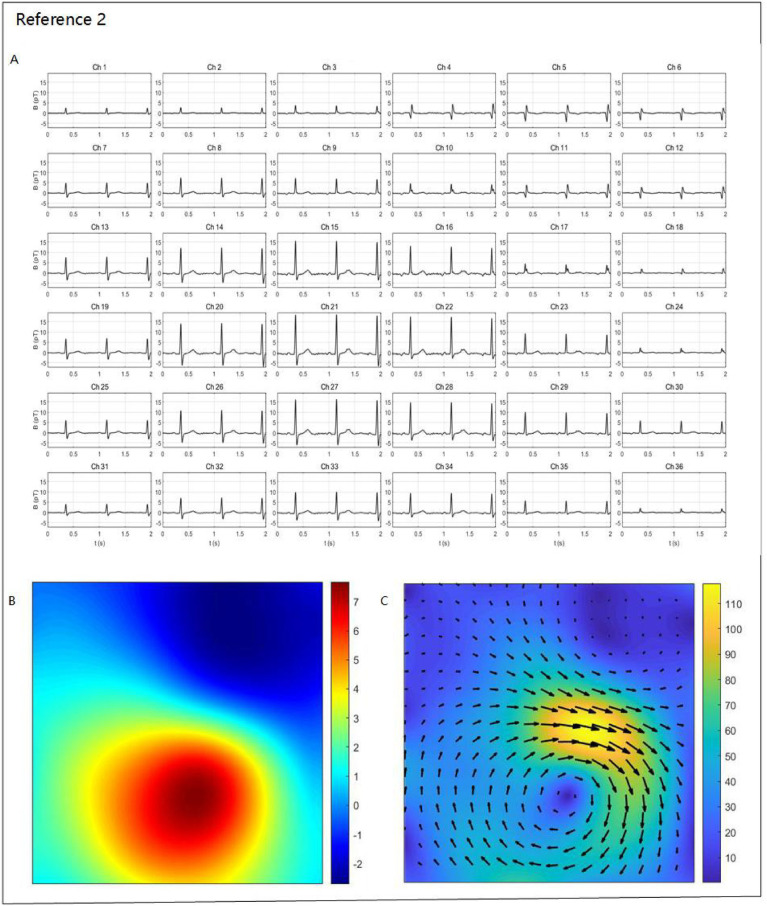


**Figure:**
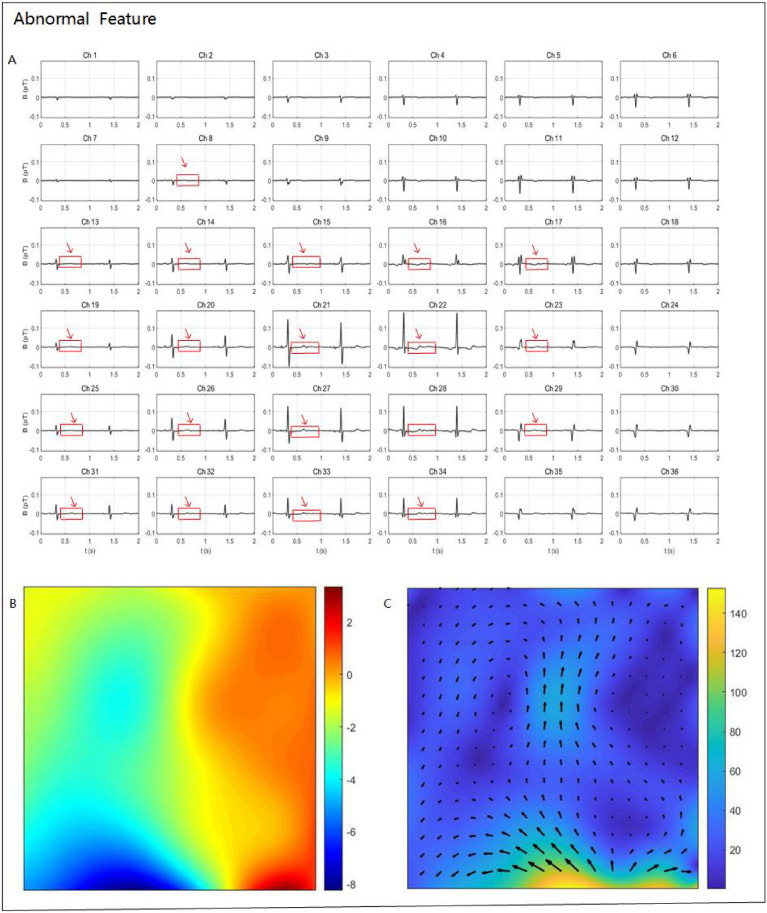


## Methods

### Data acquisition

A self-developed 36-channel SERF-MCG system to perform the MCG recordings (Figure 1). In brief, a patient lay in the supine position and the arrayed sensors on the acquisition panel positioned close to, but not in contact with chest wall. Data were then collected after positioning the laser at the top of the sternum. MCG recordings were carried out at rest for 180 s, at a sampling rate of 1,000 Hz, covering a bandwidth from DC to 100 Hz, and the operating dynamic range is ±20 nT. The raw MCG signals were initially recorded within a DC-100 Hz bandwidth without any real-time filtering. Patient recordings were performed in a standard clinical environment. The MCG system itself incorporates an integrated, multi-layer magnetic shielding cylinder that provides local active shielding, thereby eliminating the requirement for a dedicated, room-scale magnetically shielded room (MSR).

### Signal processing and averaging

Given the extremely small amplitude of MCG signals (on the order of picoteslas), signal quality optimization during preprocessing was essential. A multi-layered filtering strategy was adopted: (1) A 50 Hz low-pass filter (4th-order Butterworth, −40 dB/octave) to suppress high-frequency interference; (2) A 1–45 Hz bandpass filter (4th-order Butterworth, −40 dB/octave) to retain the main physiological components while removing baseline drifts and high-frequency noise; (3) A 50 Hz notch filter (Q = 30, −60 dB) to eliminate powerline interference. (4) The continuous MCG signals were averaged across 180 s for each subject. All filters and averaging were applied during software-based post-acquisition preprocessing.

### MCG waveform interpretation and diagnostic criteria

#### Waveform analysis and interpretation procedure

MCG waveform analysis was performed using a novel ECG-like analytical approach, which was preliminarily validated in our large-scale machine learning study (*n* = 4,500 MCG recordings, under review). To ensure objective interpretation, two senior electrocardiographers, each having undergone extensive training on a dedicated database of more than 2,000 normal MCG tracings, conducted blinded and independent assessments. Any discrepancies between their initial interpretations were resolved through a consensus process involving a third senior expert.

#### Establishment and application of normal reference ranges

Normal reference ranges for all key MCG parameters (e.g., T_amp, k1-k4_amp) were established from a large, rigorously defined healthy cohort, as detailed in Appendix Table 1 (“Establishment of Normal Reference Ranges”). These ranges define the central 95% distribution (2.5th to 97.5th percentiles) for each parameter. The normal reference ranges for 4 key ST-segment waveform parameters (K1, K2, K3, and K4, corresponding to onset, one-third, two-thirds, and terminal points of the ST segment, respectively) and for T-wave amplitude (T_amp) across all 36 channels are summarized in Table 1. The confidence intervals for the summed K1–K4 parameters across all 36 channels and other parameters are provided in Appendix Table 1.

**Table 1:** Normal reference intervals for ST-segment and T-wave parameters in 36 channels.

NO.	K1amp (pT)	K2amp (pT)	K3amp (pT)	K4amp (pT)	T_amp (pT)	NO.	K1amp (pT)	K2amp (pT)	K3amp (pT)	K4amp (pT)	T_amp (pT)
1	[0.0183, 0.0535]	[0.0173, 0.0617]	[0.0231, 0.078]	[0.0337, 0.062]	[0.523, 0.588]	2	[0.0457, 0.082]	[−0.0027, 0.0426]	[−0.052, 0.0102]	[0.066, 0.0947]	[0.523, 0.588]
3	[0.1292, 0.1765]	[−0.0516, 0.0163]	[−0.4147, −0.2923]	[0.1057, 0.1584]	[1.508, 1.640]	4	[0.2051, 0.257]	[−0.0862, 0.0072]	[−0.6663, −0.4875]	[−0.748, −0.54]	[2.657, 2.862]
5	[0.2113, 0.2639]	[−0.1146, −0.0153]	[−0.6108, −0.4331]	[−0.7616, −0.5553]	[2.512, 2.698]	6	[0.2085, 0.2575]	[−0.0924, −0.0018]	[−0.4811, −0.3225]	[−0.67 −0.479]	[2.309, 2.500]
7	[−0.0502, −0.0068]	[0.0308, 0.0972]	[0.1248, 0.21]	[0.0397, 0.0934]	[0.906, 1.003]	8	[0.0206, 0.0768]	[0.0596, 0.1439]	[0.0883, 0.1974]	[0.1069, 0.1666]	[1.129, 1.251]
9	[0.0072, 0.0758]	[−0.1037, −0.0067]	[−0.2528, −0.1054]	[0.1208, 0.1866]	[1.880, 2.077]	10	[0.1295, 0.2002]	[−0.2974, −0.1636]	[−1.1638, −0.8894]	[−1.325, −0.9852]	[4.190, 4.560]
11	[0.1552, 0.2263]	[−0.403, −0.2583]	[−1.3704, −1.0954]	[−2.0713, −1.7014]	[3.964, 4.278]	12	[0.1863, 0.2376]	[−0.1041, −0.0118]	[−0.5014, −0.346]	[−0.6629, −0.4836]	[2.062, 2.220]
13	[−0.1375, −0.0756]	[0.1265 0.235]	[0.4356, 0.593]	[0.4861, 0.6481]	[1.791, 1.957]	14	[0.1191, 0.2111]	[0.67, 0.8404]	[1.3217, 1.5875]	[1.4714, 1.7526]	[2.812, 3.076]
15	[0.172 0.2929]	[0.4898, 0.6942]	[0.9082, 1.2017]	[0.7157, 0.9252]	[2.817, 3.110]	16	[−0.0424, 0.0356]	[−0.231, −0.0977]	[−0.4047, −0.1918]	[0.0097, 0.1218]	[3.105, 3.460]
17	[0.083, 0.1528]	[−0.39, −0.254]	[−1.0642, −0.8163]	[−1.4795, −1.1716]	[3.090, 3.403]	18	[0.1653, 0.2142]	[−0.03, 0.0503]	[−0.2952, −0.1687]	[−0.3526, −0.2243]	[1.577, 1.727]
19	[−0.1953, −0.1281]	[0.1594, 0.2862]	[0.6482, 0.8504]	[0.8626, 1.0956]	[2.351, 2.550]	20	[0.1757, 0.2861]	[1.3174, 1.5471]	[2.7743, 3.1717]	[3.5524, 4.0599]	[5.029, 5.437]
21	[0.6919, 0.8551]	[2.4603, 2.789]	[4.5888, 5.1294]	[5.1375, 5.8316]	[7.195, 7.776]	22	[0.2214, 0.3636]	[1.0058, 1.2714]	[1.9623, 2.3716]	[1.027, 1.3534]	[4.241, 4.642]
23	[0.0417, 0.1114]	[0.0175, 0.1129]	[0.1103, 0.2565]	[0.0348, 0.0884]	[1.662, 1.825]	24	[0.1207, 0.162]	[0.0575, 0.1169]	[−0.0193, 0.0642]	[0.0602, 0.1048]	[0.823, 0.910]
25	[−0.1951 −0.1379]	[0.1279, 0.2329]	[0.6005, 0.7834]	[0.5552, 0.749]	[2.119, 2.311]	26	[0.0744, 0.1795]	[1.1386, 1.3526]	[2.6447, 3.029]	[3.2052, 3.7105]	[4.856, 5.246]
27	[0.4793, 0.6327]	[2.2215, 2.5268]	[4.5546, 5.0872]	[5.5046, 6.244]	[7.514, 8.091]	28	[0.2966, 0.4387]	[1.5692, 1.8715]	[3.0362, 3.5192]	[2.8203, 3.3767]	[5.647, 6.104]
29	[−0.0753, 0.0039]	[0.0869, 0.2155]	[0.5204, 0.7283]	[−0.0814, −0.0362]	[2.126, 2.325]	30	[0.0277, 0.0678]	[0.0408, 0.0909]	[0.0939, 0.1585]	[0.0364, 0.0626]	[0.636, 0.703]
31	[−0.1214, −0.0526]	[0.3329, 0.4699]	[0.9803, 1.2265]	[0.7861, 1.0384]	[2.658, 2.924]	32	[−0.0946, −0.0124]	[0.4635, 0.6222]	[1.2164, 1.4911]	[1.0888, 1.3836]	[2.972, 3.224]
33	[0.0651, 0.1735]	[0.9087, 1.1232]	[2.083, 2.451]	[2.0352, 2.4647]	[4.328, 4.688]	34	[−0.0728, 0.0189]	[0.4796, 0.6609]	[1.2023, 1.4935]	[0.8374, 1.1075]	[3.168, 3.448]
35	[−0.111, −0.0463]	[−0.0068, 0.0896]	[0.2918, 0.4431]	[0.0977, −0.0591]	[1.553, 1.702]	36	[−0.0052, 0.0383]	[−0.0046, 0.0522]	[0.1189, 0.2068]	[−0.0144, 0.0116]	[0.810, 0.904]

No.: Channel number in the multichannel MCG system. Ranges: 2.5th–97.5th percentiles.

#### Quantitative definition of abnormalities

Abnormalities were defined as quantitative deviations from the established normal reference ranges. Specifically, for any given channel, a parameter value was classified as abnormal if it fell below the 2.5th percentile or above the 97.5th percentile. For T-wave amplitude (T_amp), this percentile-based criterion (2.5th–97.5th percentile) served as the sole, objective, operational definition for quantitative classification and analysis throughout this study. This objective threshold (corresponding to a 95% reference interval) forms the basis for all subsequent diagnostic classifications.

#### Morphological definition of ischemia-related patterns

Building upon these quantitative thresholds, ischemia-related patterns were defined using specific ECG-equivalent morphological features, the validity of which was confirmed within our cohort. The operational definitions for these composite features, which integrate the abnormal parameter criteria, are provided in Table 2. It is important to note that Table 2 includes a simplified, visually-applicable rule-of-thumb for T-wave assessment (T-amplitude < 1/10 of the corresponding R-wave height) to facilitate intuitive understanding of the ischemic morphology; however, this criterion is provided for descriptive purposes only and does not supersede the primary, statistically-derived reference ranges used for case classification.

**Table 2:** Definitions and abnormality criteria for MCG morphological features.

Feature	Definition		Reference		Abnormal	
ST segment	The magnetic interval between the J point and the T-wave onset.	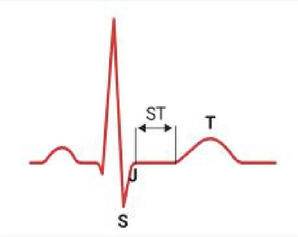	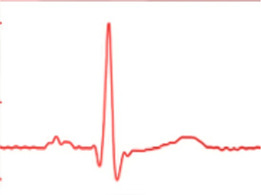	Mild upward-sloping ST elevation, with no significant horizontal or downsloping ST depression.	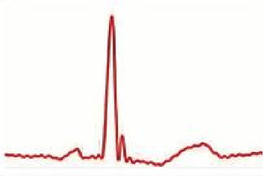	Significant depression relative to baseline, horizontal/downsloping depression, suggestive of ischemia.
T amplitude	the maximum magnetic field strength measured from the isoelectric baseline to the peak of the T wave in MCG.	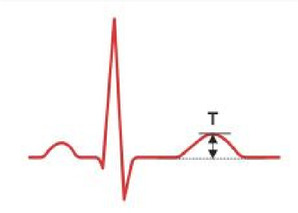	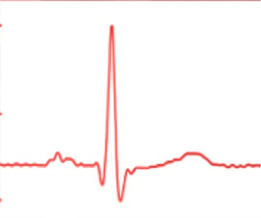	≥ 1/10 of the corresponding R-wave height in the same channel (in R-wave positive channels)	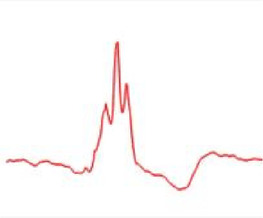	1) < 1/10 of the corresponding R-wave height in the same channel, suggestive of myocardial ischemia. 2) T-wave inversion or biphasic T waves (in R-wave positive channels).

## Case presentation

### Case 1

**Figure 3:**
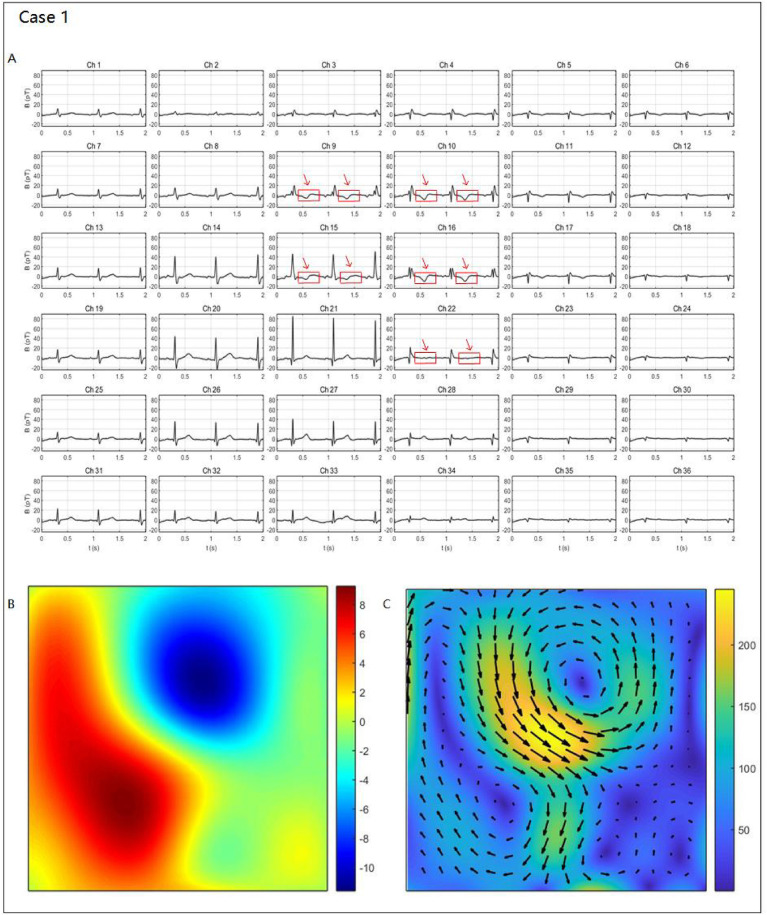
**(A)** MCG waveform maps with the ischemia features labeled; **(B)** magnetic field map at T-peak; **(C)** current density Map at T-peak magnetic field map at T-peak; **(D)** 12 lead ECG showing unremarkable; **(E)** CTA reconstruction images: the major coronary branches demonstrate a clear course, patent lumen, and no significant stenosis, plaque, or morphological abnormality is observed; **(F)** Tc-99m-sestamibi SPECT MPI showing exercise-induced perfusion defect (red arrow) in the inferior wall, and basal anterior segments of the left ventricle and the mid-septal. The color scale is optimized individually for each case to display current-flow patterns; absolute values are case-specific and should be interpreted in conjunction with the spatial distribution and temporal changes of the currents.

A 45-year-old man presented to EDs on April 15, 2024, with recurrent episodes of non-radiating angina pectoris. He denied a history of hypertension, diabetes mellitus, smoking or significant alcohol consumption. Vital signs were temperature 36.5°C heart rate 70 beats per minute (bpm), respiratory rate 16 breaths per minute (bpm), and blood pressure 108/74 mmHg. Initial ECG was unremarkable (Figure 3D). Standard laboratory tests, including complete blood count and basic metabolic panel were within normal ranges. D-dimer level was also within the normal range. Cardiac troponin I (cTnI) level was 0.01 μg/L, which was below the gender-specific normal cutoff of 0.023 μg/L. Additionally, the creatine kinase-myocardial band (CK-MB) level was 1 μg/L (normal cutoff: 5 μg/L), and the myoglobin level was 48 μg/L (normal cutoff: 112 μg/L). Other initial assessments, such as echocardiography, revealed no remarkable findings.

**Figure:**
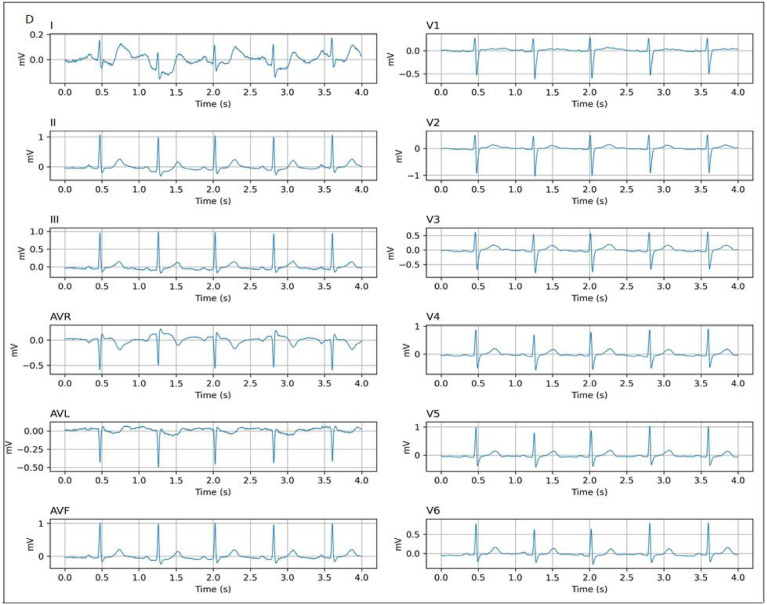


**Figure:**
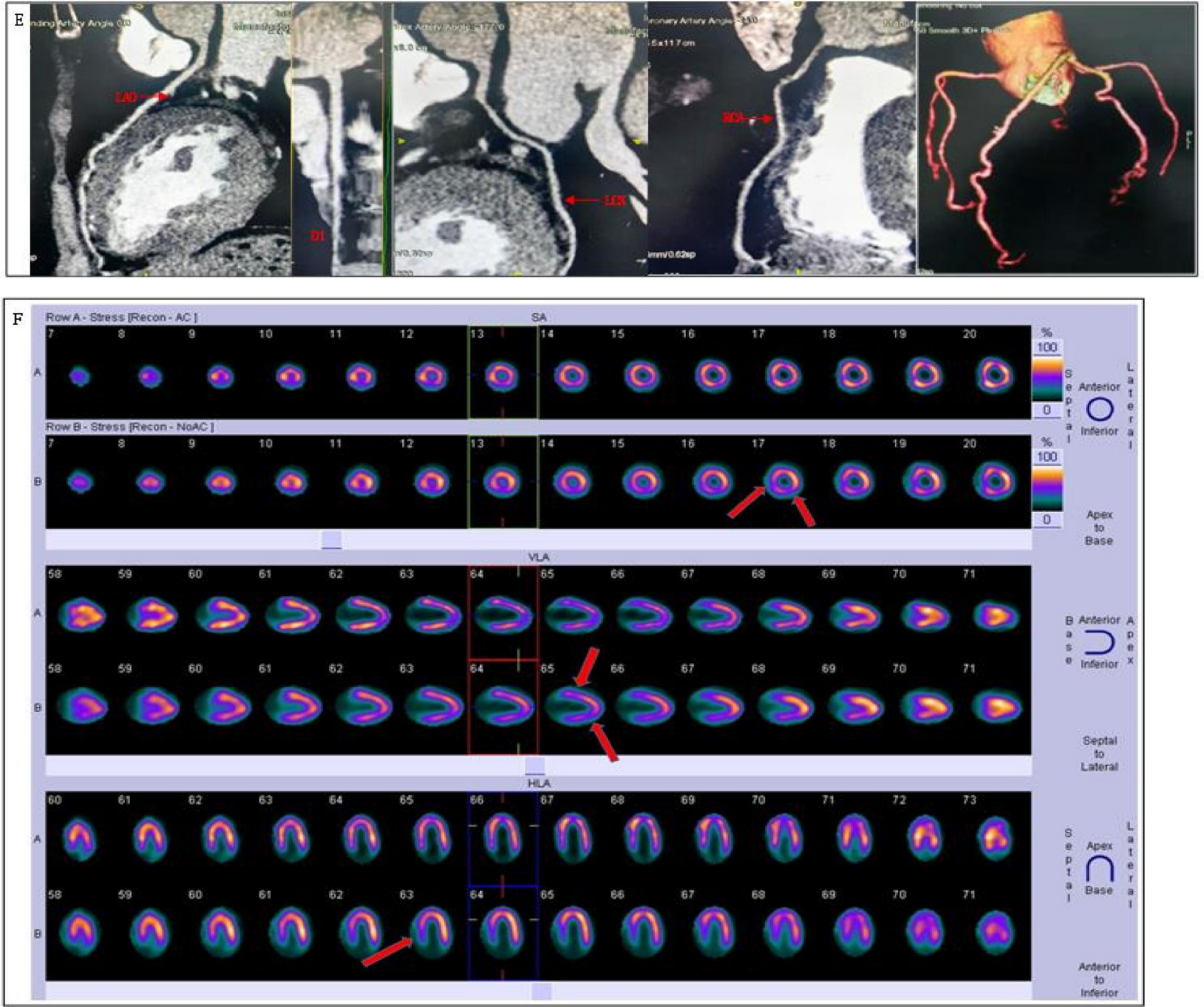


CCTA performed on the same day (April 15, 2024) (Figure 3E). On the same day, the patient underwent 36-channel SERF-MCG, which revealed a distinct pattern of electromagnetic abnormalities. Quantitative analysis of all MCG parameters is detailed in Table 4. Specifically, ST-segment depression, flattening, or T-wave inversion was observed in channels 9, 10, 15, 16, 22, 24, 25, 28, 29, and 30, characterized by reduced K1-K4 amplitudes (indicated by arrows in Table 3). Notably, the amplitude in four of these channels was more than tenfold below the lower limit of the normal reference range (Table 3). Manual review confirmed significant ST-T segment depression and flattening in channels 9, 10, 15, 16, and 22, findings that were highly consistent with the automated detection results (Figure 3A). Meanwhile, abnormal magnetic field distribution was observed at both the positive and negative magnetic poles at the T-wave peak, accompanied by current changes in the pseudo current density map at this same time point (Figures 3B,C).

**Table 3:** K1-4 amp, and T_amp for case 1 (36-channel MCG).

NO.	K1amp (pT)	K2amp (pT)	K3amp (pT)	K4amp (pT)	T_amp (pT)	NO.	K1amp (pT)	K2amp (pT)	K3amp (pT)	K4amp (pT)	T_amp (pT)
1	0.524	1.269	2.041	2.718	2.745	2	0.176	0.373	0.52	0.552	0.551
3	0.08	−0.259	−0.356	−0.305	2.82	4	0.145	0.009	−0.036	−0.028	4.72
5	0.327	0.093	−0.074	−0.114	3.024	6	0.229	0.048	−0.069	−0.086	3.072
7	0.285	1.329	2.36	3.29	3.312	8	0.592	1.496	2.81	3.857	3.902
9	−1.062↓↓↓↓	−1.669↓↓↓↓	−3.799↓↓↓↓	−6.291↓↓↓↓	6.325	10	−0.268	−1.53↓	−5.426↓	−9.842↓↓	9.915
11	−0.089	−0.677	−2.375	−4.453	4.505	12	0.119	0.013	0.005	0.069	1.689
13	−0.207	−0.427	−0.46	−0.206	4.247	14	1.66	3.168	4.69	5.903	5.965
15	−1.174↓↓	−0.174↓	−3.17↓	−6.169↓↓	4.029	16	−0.361↓↓	−1.464↓↓	−5.461↓↓↓	−9.474↓↓↓↓	10.093
17	0.005	−0.635	−2.196	−4.135	4.192	18	0.147	0.012	−0.018	0.034	1.529
19	0.068	0.744	2.508	3.909	3.937	20	1.614	3.364	6.179	8.121	8.171
21	2.001	5.651	4.748	0.111	6.507	22	0.007↓	0.021↓	0.014↓	0.079↓	0.221
23	0.244	0.096	−0.069	−0.091	1.427	24	−0.06	−0.118↓	−0.222↓↓	−0.239↓	1.056
25	−0.262↓	−0.139↓	−0.098↓	−0.098↓	2.597	26	−0.013	0.992	3.507	6.571	6.625
27	0.12	1.067	3.792	7.755	7.822	28	0.157	0.039↓	−0.307↓	−0.379↓	3.895
29	−0.045	−0.333↓	−0.273↓	−0.032	0.257	30	0.099	−0.112↓	−0.181↓	−0.183↓↓	0.223
31	0.06	0.635	2.266	4.135	4.159	32	0.521	0.644	1.911	3.237	3.257
33	0.1	−0.014	−0.123↓	−0.344↓	3.739	34	−0.385↓	−0.164↓	−0.133↓	−0.287↓	2.017
35	−0.11↓	−0.138↓↓↓↓	0.334	0.024	0.397	36	0.102	0.078	0.056	0.066	0.252

↓, ↓↓, ↓↓↓, ↓↓↓↓ indicate values reduced to 1–5, 5–10, 10–15, and ＞15 times the reduced confidence interval limit, respectively.

To further evaluate the presence of myocardial ischemia, the patient underwent a stress Tc-99m-sestamibi single-photon emission computed tomography myocardial perfusion imaging (SPECT MPI) the following day (April 16, 2024) (Figure 3F). SPECT MPI demonstrated mild perfusion abnormalities in the inferior wall, and basal anterior of the left ventricle, and the mid-septal segment (summed stress score [SSS] = 1; Figure 3F). Additionally, mild to moderate hypokinesis was observed in the apical anterior segment of the left ventricle and the ventricular septum. The concordance of these functional imaging findings with the distinct MCG abnormalities was consistent with, but not definitive for, the presence of underlying myocardial ischemia in the absence of obstructive CAD.

Based on the integrated findings, a working diagnosis of suspected ANOCA was made. Following the diagnostic work-up, the patient was started on guideline-directed medical therapy for suspected microvascular angina, including a beta-blocker and a long-acting nitrate. At a 3-month telephone follow-up, he reported a significant reduction in the frequency of his angina episodes and expressed satisfaction with the symptomatic improvement.

### Case 2

**Figure 4:**
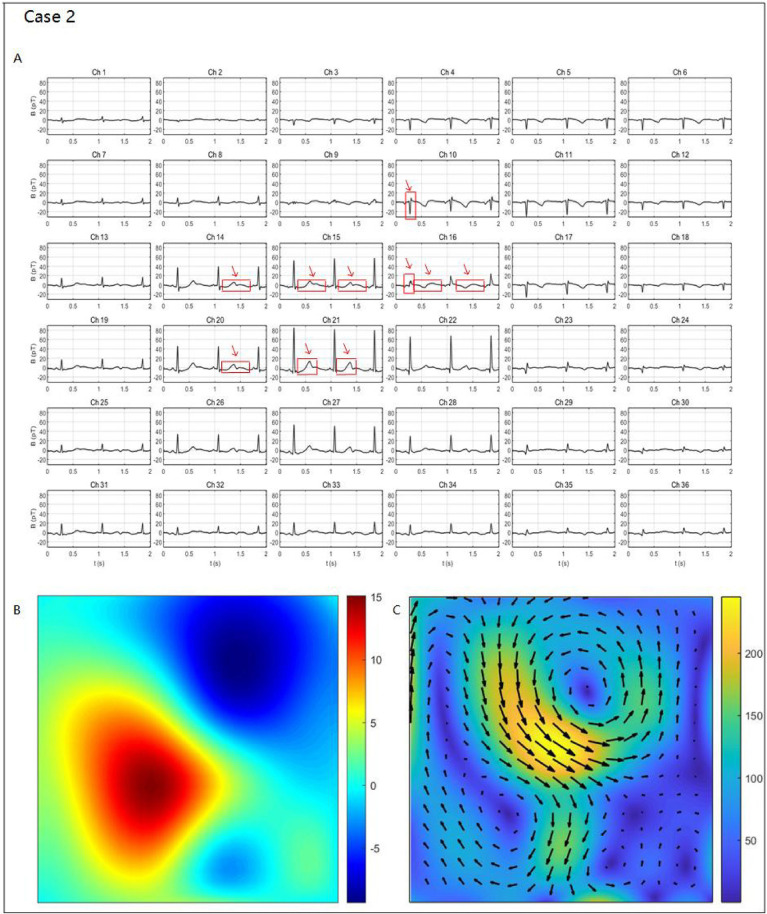
**(A)** MCG waveform maps with the ischemia features labeled; **(B)** magnetic field map at T-peak; **(C)** Pseudo current density Map at T-peak magnetic field map at T-peak; **(D)** 12 lead ECG showing unremarkable; **(E)** CTA reconstruction images: the major coronary branches demonstrate a clear course, patent lumen, and no significant stenosis, plaque, or morphological abnormality is observed; **(F)** Tc-99m-sestamibi SPECT MPI showing rest perfusion defect (red arrow) in the anterior wall segments of the left ventricle. The color scale is optimized individually for each case to display current-flow patterns; absolute values are case-specific and should be interpreted in conjunction with the spatial distribution and temporal changes of the currents.

A 53-year-old woman presented for evaluation of recurrent angina pectoris. She had a history of diabetes mellitus and hyperlipidemia, but denied hypertension, smoking, or alcohol consumption. Vital signs were as follows: temperature 36.2°C, heart rate 85 beats per minute (bpm), respiratory rate 20 breaths per minute (bpm), and blood pressure 135/72 mmHg. ECG was unremarkable and standard laboratory tests, including complete blood count and basic metabolic panel were within normal ranges. D-dimer was also within the normal range. High sensitivity troponin (hsTn) level was 1.71 ng/L, which stayed below the gender-specific normal cutoff of 11.6 ng/L. Additionally, the CK-MB level was 3.07 μg/L (normal cutoff: 5 μg/L). Other initial assessments, such as echocardiogram, revealed no remarkable findings. CCTA scan showed no abnormalities (Figure 4E). The same day, 36-channel SERF-MCG was performed. The analysis revealed a distinct pattern of abnormalities in the cardiac magnetic signal. Quantitative data for all MCG parameters are summarized in Table 4. Specifically, ST-segment depression, flattening, or T-wave inversion was observed in 22 channels, characterized by reduced amplitudes of the K1–K4 waveform points (as indicated by arrows in Table 4). Notably, the amplitude in 12 of these channels fell more than tenfold below the lower limit of the normal reference range. Manual review confirmed prominent ST-T segment depression and flattening in channels 14, 15, 16, 20, and 21, along with poor R-wave progression in channel-10 (6.005 pT; reference: 9.1424–9.7753 pT). These findings were highly consistent with the automated detection results (Figure 4A). Poor R-wave progression in channel-10. Meanwhile, abnormal magnetic field distribution was observed at both the positive and negative magnetic poles at the T-wave peak, accompanied by current changes in the pseudo current density map at this same time point (Figures 4B,C).

**Figure:**
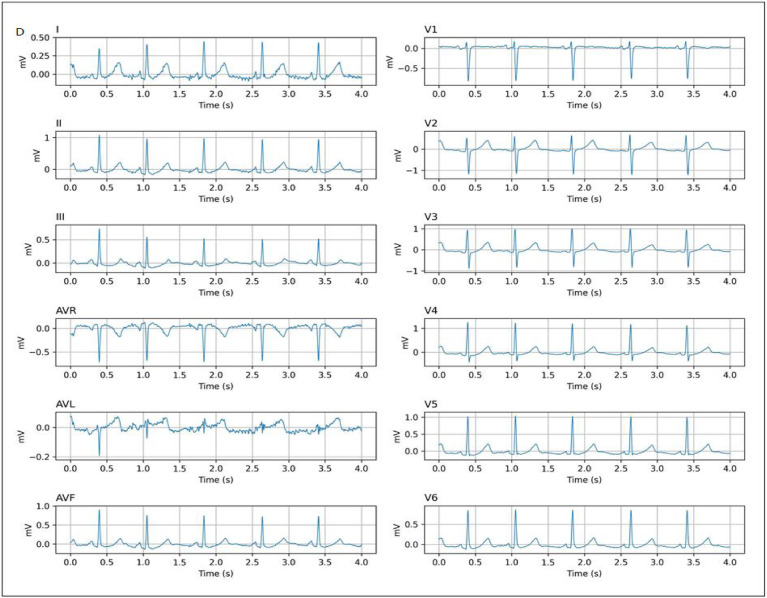


**Figure:**
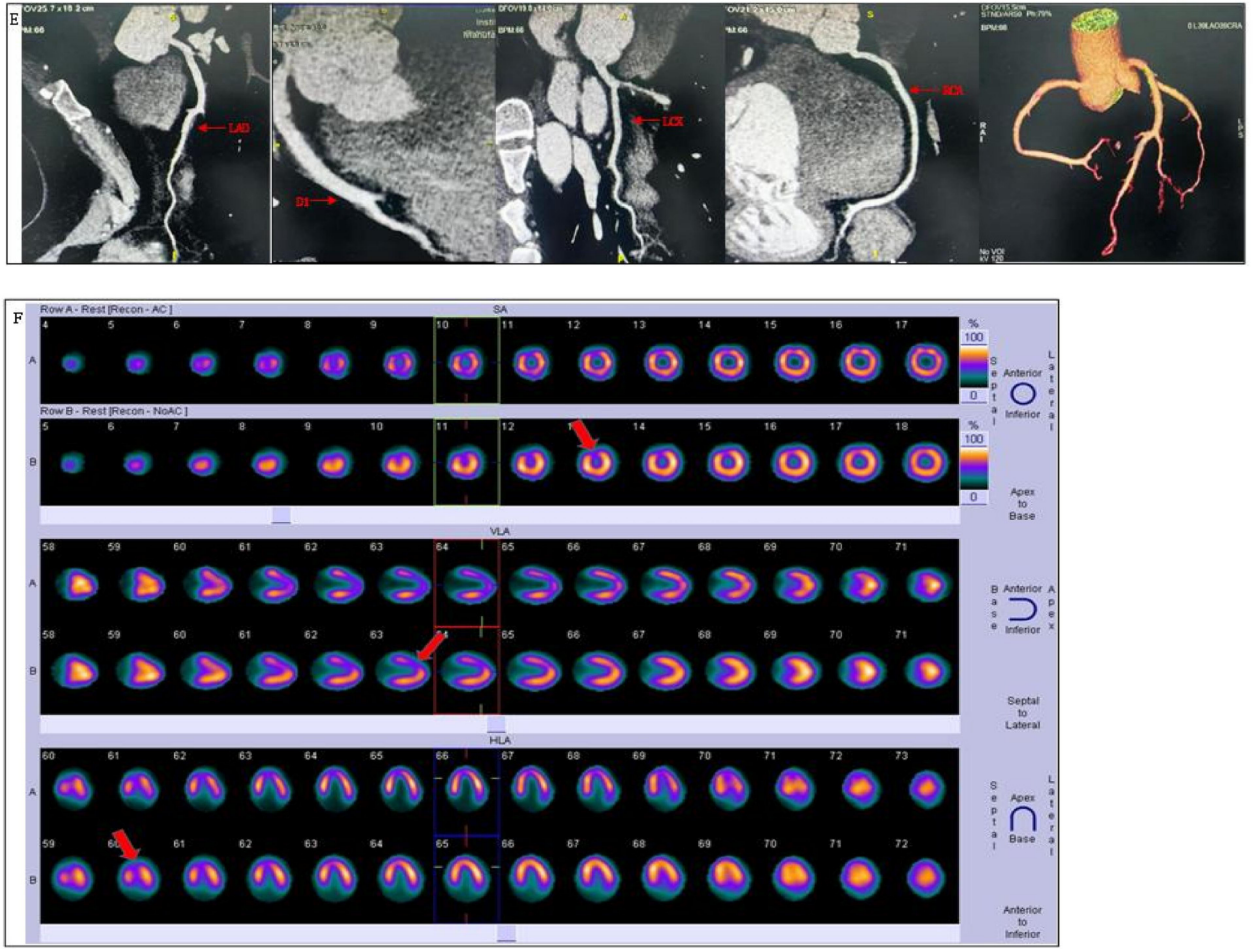


**Table 4:** K1-4 amp, and T_amp for case 2 (36-channel MCG).

NO.	K1amp (pT)	K2amp (pT)	K3amp (pT)	K4amp (pT)	T_amp (pT)	NO.	K1amp (pT)	K2amp (pT)	K3amp (pT)	K4amp (pT)	T_amp (pT)
1	0.542	0.771	1.272	1.652	1.69	2	0.453	0.49	0.433	0.345	0.357
3	0.858	0.844	0.866	0.878	2.946	4	0.388	−1.097↓↓↓	−3.721↓	−6.029↓↓	6.107
5	−0.183↓	−1.6↓↓↓	−3.421↓	−5.017↓↓	5.082	6	−0.255↓	−1.852↓↓↓↓	−3.62↓	−5.215↓↓	5.279
7	0.23	0.799	1.284	1.886	1.935	8	0.915	1.307	2.171	2.934	3.044
9	0.738	0.561	0.513	0.564	2.719	10	−0.389↓	−2.667↓↓	−6.418↓	−9.113↓↓	9.218
11	−0.419↓	−2.609↓↓	−5.205↓	−7.429↓	7.518	12	−0.089↓	−1.124↓↓	−2.191↓	−3.243↓	3.282
13	0.476	1.545	2.583	3.583	3.646	14	−1.206↓↓↓	−1.754↓	−1.578↓	0.613↓	8.44
15	−1.146↓↓	−1.783↓	−1.615↓	0.563	7.374	16	0.203	−0.723↓	−2.747↓↓	−4.612↓↓↓	4.649
17	−0.595↓↓	−2.245↓	−3.777↓	−4.923↓	4.977	18	−0.071↓	−0.796↓↓↓↓	−1.649↓	−2.528↓↓	2.571
19	0.427	1.611	2.754	3.79	3.86	20	2.422	5.014	7.456	9.817	9.986
21	2.094	6.073	10.347	14.687	14.959	22	2.004	4.38	6.169	−0.027↓	6.739
23	−0.381↓↓↓	−0.753↓↓↓↓	−1.008↓↓↓	−1.144↓↓↓↓	1.167	24	−0.046↓	−0.324↓↓	−0.765↓↓↓	−1.313↓↓↓↓	1.35
25	0.814	2.117	1.967	−0.208↓	2.622	26	2.846	5.876	5.218↓	−0.059	7.057
27	3.334	7.648	7.748	−0.22↓	9.899	28	1.641	4.017	4.738	−0.205	5.374
29	0.087↓	0.523	1.058	−0.97	1.067	30	0.207	0.233	0.196	0.073	0.834
31	1.315	3.224	3.001	−0.232↓	4.029	32	0.725	1.891	2.014	−0.207↓	2.408
33	−1.26↓	−0.351↓	3.053	−0.281↓	3.947	34	0.639	2.145	2.49	−0.53↓	2.903
35	0.09	0.524	0.839	−0.155↓	0.842	36	0.064	0.296	0.607	−0.299↓	0.645

↓, ↓↓, ↓↓↓, ↓↓↓↓ indicate values reduced to 1–5, 5–10, 10–15, and ＞15 times the reduced confidence interval limit, respectively.

Given the patient's multiple cardiovascular risk factors, the cardiology team recommended myocardial perfusion imaging for further evaluation. Subsequent rest SPECT MPI, performed the following day, revealed mild myocardial ischemia in the anterior wall segments of the left ventricle (Figure 4F). It is noted that a resting defect, in the context of normal coronary arteries, preserved systolic function, and negative cardiac biomarkers, has a broad differential diagnosis and its direct attribution to active myocardial ischemia is uncertain. Concurrently, mild to moderate hypokinesis was identified in the anterior segment of the left ventricle. However, the presence of distinct MCG waveform abnormalities, combined with this objective (though non-specific) imaging finding, heightened the clinical suspicion for an underlying ischemic etiology.

Based on the integrated findings, a working diagnosis of suspected ANOCA. The patient was started on guideline-directed medical therapy, including (metoprolol succinate 23.75 mg once daily) and a statin (atorvastatin 20 mg once daily). At the 3-month follow-up, she reported a significant reduction in angina frequency. Further invasive or stress perfusion testing was not pursued during the documented clinical course.

### Case 3

**Figure 5:**
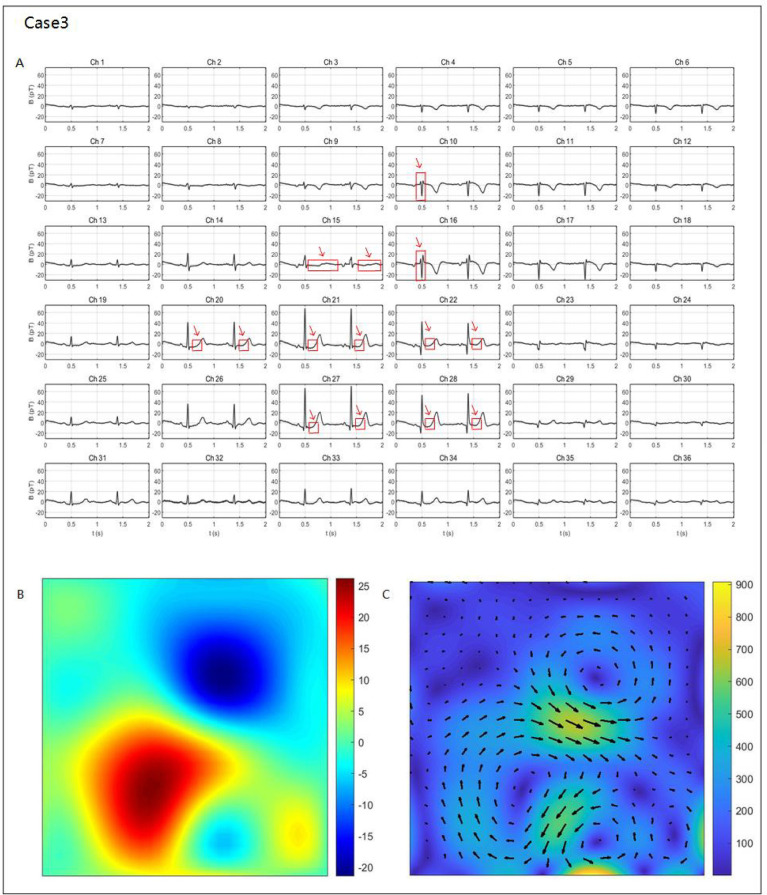
**(A)** MCG waveform maps with the ischemia features labeled; **(B)** Magnetic field map at T-peak; **(C)** Pseudo Current Density Map at T-peak Magnetic field map at T-peak; **(D)** 12 lead ECG showing nonspecific ST-T wave changes; **(E)** CTA reconstruction images: the major coronary branches demonstrate a clear course, patent lumen, and no significant stenosis, plaque, or morphological abnormality is observed; **(F)** Tc-99m-sestamibi SPECT MPI showing rest perfusion defect (red arrow) in the anterior apical segment and mid-anterior segment of the left ventricle. Note: The color scale is optimized individually for each case to display current-flow patterns; absolute values are case-specific and should be interpreted in conjunction with the spatial distribution and temporal changes of the currents.

A 56-year-old female presented to the ED with a chief complaint of chest pain. The pain was in the sternal region, characterized as squeezing and pressure-like, radiating to the hypogastrium and pharynx, exacerbated by exertion, and lasting approximately one hour. She reported recurrent chest pain over the last 8 month. On presentation, vital signs were temperature 36.5°C, heart rate 69 bpm, respiratory rate 20 bpm, blood pressure 107/66 mmHg, and oxygen saturation 98% on room air. Physical examination revealed no abnormal findings, and she denied a history of alcohol consumption or tobacco use. ECG showed nonspecific ST-T wave changes (Figure 5A). cTnI level was 0.001 μg/L, which stayed below the gender-specific normal cutoff of 0.023 μg/L. Additionally, the CK-MB level was 1.4 μg/L (normal cutoff: 5 μg/L), and the myoglobin level was 10.3 μg/L (normal cutoff: 112 μg/L). Other initial assessments, such as echocardiography, revealed no remarkable findings. CCTA performed on the same day showed no significant coronary artery plaques or stenosis (Figure 5E).

**Figure:**
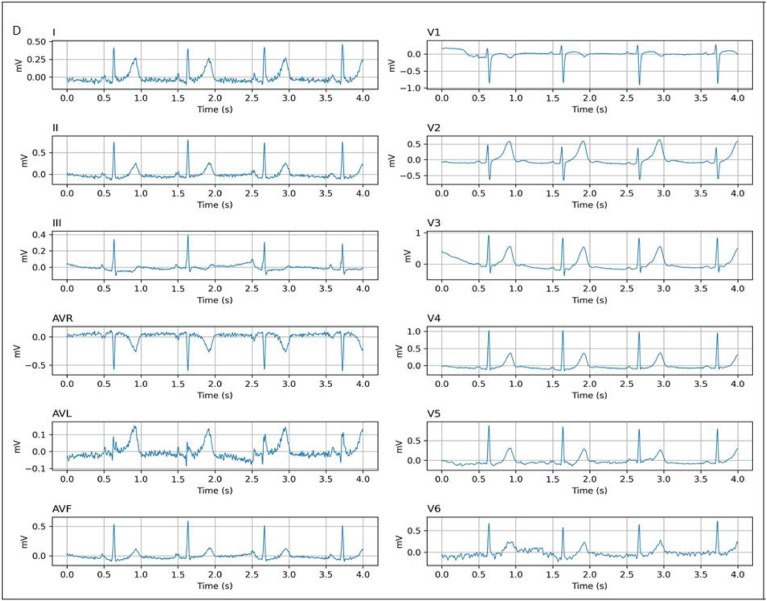


**Figure:**
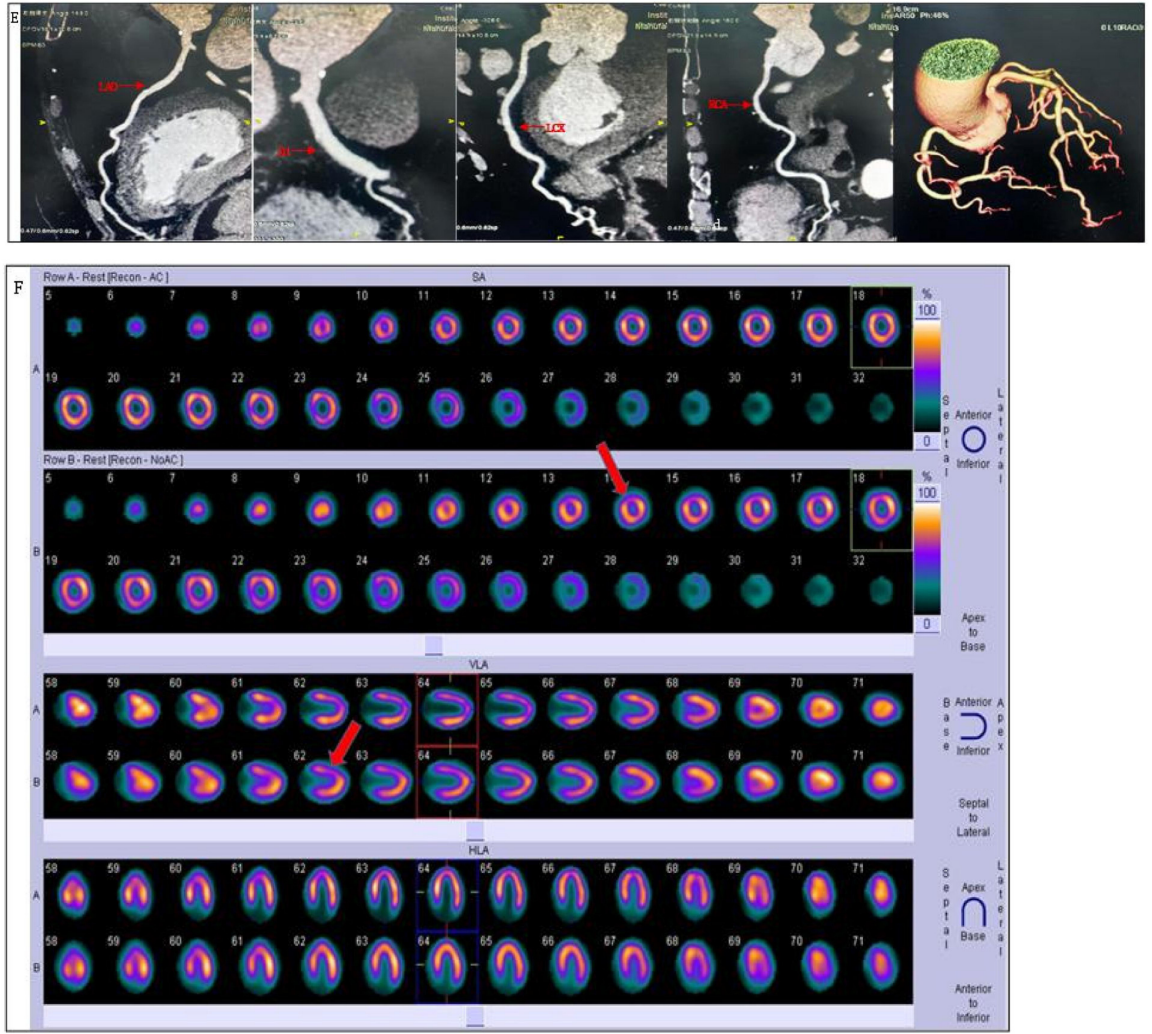


The same day, the patient underwent 36-channel SERF-MCG. The analysis identified a distinct pattern of abnormalities in the cardiac magnetic signal. Quantitative data for all MCG parameters are summarized in Table 5. ST-T segment depression, flattening, or inversion was observed in a total of 24 channels, characterized by reduced amplitudes at the K1–K4 waveform points (as indicated by arrows in Table 5). Notably, the amplitude in 18 of these channels was more than tenfold below the lower limit of the normal reference range. Manual review confirmed prominent ST-T segment depression and flattening in channels 15, 16, 20, 21, 22, 27, and 28. Additionally, poor R-wave progression was observed in channel-10 (7.418 pT; normal reference range: 9.1424–9.7753 pT) and channel-16 (10.685 pT; normal reference range: 14.3657–15.5294 pT). These findings were highly consistent with the automated detection results (Figure 5A). Meanwhile, abnormal magnetic field distribution was observed at both the positive and negative magnetic poles at the T-wave peak, accompanied by current changes in the pseudo current density map at this same time point (Figures 5B,C).

**Table 5:** K1-4 amp, and T_amp for case 3 (36-channel MCG).

NO.	K1amp (pT)	K2amp (pT)	K3amp (pT)	K4amp (pT)	T_amp (pT)	NO.	K1amp (pT)	K2amp (pT)	K3amp (pT)	K4amp (pT)	T_amp (pT)
1	0.103	−0.082↓↓	−0.386↓↓↓	−0.015↓	0.369↓	2	0.431	0.023	−1.189↓↓↓↓	0.112	1.374
3	0.815	−0.342↓↓	−4.097↓↓	0.329	4.285	4	0.885	0.967	0.85	0.662	6.539
5	0.851	0.833	0.716	0.487	5.087	6	−0.204↓	−1.711↓↓↓↓	−4.344↓↓	−6.287↓↓	6.359
7	−0.045	0.237	0.498	0.69	0.701↓	8	0.57	0.069↓	−0.081↓↓	−0.454↓	0.024↓
9	1.435	1.339	1.115	0.935	6.999	10	0.792	−1.403↓	−7.934↓↓	−14.947↓↓	15.07
11	−0.473↓	−2.808↓	−6.99↓	−9.912↓	10.013	12	−0.015↓	−1.068↓↓	−2.689↓	−3.929↓	3.982
13	0.886	0.952	0.846	0.685	1.991	14	0.388	0.395	0.346	0.264	0.404
15	0.188↓	0.195↓	0.146↓	0.164↓	1.706	16	1.356	−0.774↓	−8.269↓↓↓↓	−17.053↓↓↓↓	17.189
17	1.758	1.546↓↓	1.27↓	0.775↓	11.134	18	0.84	0.732↓↓↓↓	0.638↓	0.586↓	3.63
19	0.363↓	1.771	3.25	4.091	4.14	20	−1.699↓↓↓	1.231	9.927	−0.22↓	12.859
21	−2.799↓↓	0.312↓	17.834	22.565	22.771	22	−1.719↓↓	1.357	12.646	−0.32↓	13.353
23	−0.023↓	0.103	0.909	0.268	1.209	24	0.661	0.422	0.521	0.525↓↓↓↓	1.266
25	0.251	1.523	2.891	3.674	3.71	26	1.283	5.078	9.54	12.273	12.378
27	−2.289↓↓	0.012↓	17.3	22.957	23.221	28	−2.312↓↓	0.023↓	10.763	14.002	14.14
29	1.821	4.524	3.896	−0.112	5.071↓	30	−0.054↓	−0.243↓↓	0.208	0.192	0.523↓
31	−1.664↓↓↓	−1.431↓↓	−1.45↓	−0.659↓	6.615	32	−0.907↓↓	−0.421↓	3.347	−0.454↓	3.76
33	−1.392↓↓↓↓	−0.368↓	6.966	−0.55↓	7.927	34	−1.506↓↓↓↓	−0.04↓	7.499	−0.452↓	8.334
35	1.097	3.018	2.52	−0.272↓	3.368	36	1.284	2.722	2.395	0.104	2.975

↓, ↓↓, ↓↓↓, ↓↓↓↓ indicate values reduced to 1–5, 5–10, 10–15, and ＞15 times the reduced confidence interval limit, respectively.

Given the patient's severe ischemic symptoms, the cardiology team recommended myocardial perfusion imaging for further evaluation. Considering the symptom severity, resting SPECT MPI was subsequently performed the following day instead of the high-risk stress SPECT. This revealed a fixed perfusion defect in the apical anterior segment and mid-anterior segment of the left ventricle (Figure 5F). As in previous cases, the specific etiology of a resting perfusion defect in this clinical context remains to be definitively established. Nevertheless, the combination of typical symptoms, abnormal functional findings on both MCG and SPECT MPI, and the exclusion of obstructive CAD formed a cohesive clinical picture.

A working diagnosis of suspected ANOCA was made, with coronary microvascular dysfunction considered a leading etiology. Treatment with anti-anginal therapy (including a calcium channel blocker and nitrates) was initiated. At a 4-week follow-up, the patient reported a noticeable decrease in the intensity and frequency of her chest pain episodes.

### Differential diagnosis

The patients in this series presented with typical angina, had obstructive coronary artery disease ruled out by CCTA, yet showed evidence of myocardial ischemia on functional testing. Establishing the working diagnosis of “suspected ANOCA” requires a systematic evaluation to exclude other conditions that can manifest with a similar clinical profile.

Key considerations include coronary artery vasospasm, a well-established cause of angina in the absence of obstructive CAD. As intracoronary acetylcholine provocation testing was not performed, vasospastic angina cannot be definitively excluded in these cases. Therefore, it remains an important diagnostic consideration within the broad spectrum of suspected ANOCA.

Structural heart diseases were also evaluated. Conditions such as hypertrophic cardiomyopathy or inflammatory sequelae (e.g., post-myocarditis) can present with angina, ECG changes, and regional wall motion abnormalities. In our patients, transthoracic echocardiography did not reveal significant ventricular hypertrophy or structural anomalies, and cardiac biomarkers were persistently negative. These findings make significant structural heart disease less probable.

Gastroesophageal reflux disease (GERD) is a common mimicker of cardiac pain. Our patients' symptoms were closely tied to exertion and showed initial response to anti-anginal therapy, supporting a cardiac origin. Furthermore, the classic features of reflux-related chest pain (postprandial worsening, retrosternal burning, association with recumbency) were not prominent in these cases, and no patient reported typical reflux symptoms such as heartburn or acid regurgitation. Though coexistence with GERD is possible, the temporal relationship with exertion and the objective functional abnormalities prioritize the evaluation of a cardiac etiology.

Spontaneous coronary artery dissection (SCAD) is another critical consideration, particularly in the female patients (Cases 2 and 3), as it predominantly affects middle-aged women. SCAD can present with acute chest pain, myocardial ischemia, and normal or near-normal coronary angiograms, thereby mimicking the ANOCA phenotype. In our cases, the absence of established high-risk features (such as a history of fibromuscular dysplasia, peripartum state, or connective tissue disease), coupled with the lack of characteristic angiographic findings (e.g., arterial wall haematoma) on high-quality CCTA, makes SCAD a less likely etiology. Its consideration and exclusion were based on the integration of available clinical and anatomical data.

After this differential diagnostic process, the integrated findings—typical symptoms, non-obstructive coronaries, and objective functional abnormalities—most strongly support a working diagnosis of suspected ANOCA. It is crucial to emphasize that, according to current guidelines, the definitive diagnosis and phenotyping of ANOCA endotypes (distinguishing microvascular dysfunction from vasospasm) require invasive coronary function testing. This represents a recognized limitation of our retrospective study and precisely defines the necessary direction for future validation research.

## Discussion

Patients presenting with angina pectoris and found to have non-obstructive coronary arteries on imaging constitute a distinct clinical phenotype. The underlying cause of this presentation is often suspected to be ANOCA, which requires further functional testing for definitive diagnosis (7). In clinical practice, the initial evaluation of these patients often relies on a sequence of tests, each with its own practical constraints. Resting ECG has limited value in diagnosing myocardial ischemia in this context. Although stress testing (exercise or pharmacological) combined with imaging (echocardiography or perfusion scintigraphy) can provide functional evidence, its implementation is often hindered by suboptimal patient suitability, concerns about radiation exposure, procedural risks, or resource availability. This frequently results in a diagnostic gap where anatomical assessment (via CCTA or invasive angiography) rules out obstructive disease, but objective evidence of ischemia—crucial for confirming INOCA and guiding therapy—remains elusive. This gap underscores the need for a diagnostic tool that can seamlessly integrate into the early assessment workflow, providing functional information that is both sensitive to microvascular disturbances and practical for routine use. Developing such a method is therefore critical for improving the evaluation and management of patients with suspected ANOCA.

In fact, when the ECG and cardiac enzyme patterns are not yet diagnostic, the theoretical advantage of MCG lies in its potential for detecting myocardial ischemia at an early stage, owing to its more comprehensive biomagnetic electrophysiological assessment. MCG can non-invasively detect electrogenic phenomena at the cellular and even subcellular levels (8, 9), making it an emerging novel method for detecting cardiac ischemia. Foundational theoretical and experimental research indicates that early-stage ischemia induces changes in myocardial electrophysiological properties that are detectable by MCG but not by ECG. This is because MCG is more sensitive to tangential currents, curl currents, transmural current flow, and closed-loop currents—all of which are invisible to ECG (10–14). Moreover, as noted earlier, cardiac magnetic fields are not attenuated or distorted by differences in the conductivity of body tissues or fluids, and MCG does not require skin-electrode contact. More importantly, as experimentally demonstrated by Cohen using direct current (DC)-MCG measurements, ischemia-related diastolic injury currents can be detected (15). Such “silent” abnormal electrotonic currents typically arise at the border zone of an ischemic region and may be arrhythmogenic if they reach the excitability threshold of surrounding normal tissue (16). Thus, MCG theoretically offers the capability to detect subtle, preclinical electrical abnormalities that may be associated with ischemic or other pathophysiological processes (17). The waveform patterns observed in our patient cohort—such as ST-T segment changes and altered R-wave progression—are compatible with the types of electrophysiological disturbances that this technique is proposed to capture. This provides a rationale for further investigating MCG in the evaluation of patients with angina and non-obstructive coronary arteries.

Like other currently used noninvasive functional tests, MCG does not directly visualize coronary arteries and is therefore classified as a functional, rather than anatomic, test (17). Previous literature has explored the diagnostic performance of MCG (18, 19). In this case series, we observed that resting MCG detected distinct electrophysiological abnormalities in symptomatic patients with non-obstructive coronary arteries. Importantly, this assessment was achieved without the need for exercise or pharmacologic stress, and it involves no ionizing radiation.

While the diagnostic potential of MCG is clear, the interpretation of its two-dimensional magnetic field and current density maps in previous studies can be challenging in practice, primarily due to the sometimes unclear etiology of magnetic pole rotations and visual variations. This warrants a cautious approach to interpreting these maps in the clinical setting. In this study, we describe distinct MCG waveform characteristics observed in patients with angina and non-obstructive coronary arteries, based on a specific set of pathological waveform characteristics. This work extends prior research on MCG in non-obstructive CAD and microvascular ischaemia. These include distinct ST-T segment deflections induced by injury currents secondary to myocardial ischemia, and poor R-wave progression resulting from reduced depolarization capacity and conduction delay in ischemic myocardial cells—changes that create “electrically silent regions”. These regions attenuate the depolarizing currents critical for normal R-wave progression, thereby disrupting the physiological pattern of gradually increasing R-wave amplitude across the precordial channels. Consequently, the ischemia-induced progressive decrease in R-wave amplitude manifest as well-recognizable changes in MCG channels 10 and 16. These MCG morphological features, which best discriminate between patients with angina and non-obstructive coronary arteries and healthy controls, may be attributable to vortex currents—currents not detectable by ECG. Notably, MCG waveform abnormalities represent early, suggestive signs of silent myocardial ischemia. Such morphological changes are common in our patient cohort, underscoring MCG's potential utility for detecting microcirculatory ischemic events from onset. In contrast to healthy individuals, the studied patients exhibit a transition from a stable dipolar configuration of the magnetic field map to a nondipolar pattern at the T-wave apex; concurrent changes in the pseudo-current density map further support our morphological interpretation of these waveform abnormalities.

Thus, our findings suggest that MCG not only holds potential for mechanistically differentiating microvascular dysfunction from non-ischemic conditions, but also provides clinical evidence supporting the further investigation of MCG waveform mapping in detecting subtle cardiac ischemia. The observed patterns—such as specific ST-T segment changes and altered R-wave progression—represent objective, electrophysiological correlates of the patient's symptomatic state, which were not evident on conventional resting ECG. Coupled with its non-invasive, stress-free, and radiation-free nature, these observations support MCG as a candidate tool warranting further investigation for the functional assessment of patients with chest pain and non-obstructive coronary arteries. Its ability to provide such functional information at rest could position MCG uniquely in the early diagnostic workflow, potentially helping to identify which patients with non-obstructive anatomy are most likely to benefit from more advanced or invasive testing. This study contributes to the translation of experimental MCG measurements towards clinical application. Concurrently, it highlights that ECG and CAG may underestimate the extent and severity of myocardial ischemia. Waveform morphological features constitute the mainstay of ECG for detecting myocardial ischemia—and this fundamental principle applies equally to MCG. Our results, consistent with prior studies, indicate that MCG provides diagnostic information in this challenging patient population (18, 20, 21). This underscores MCG's potential for broader clinical application in the assessment of suspected microvascular ischemia. In fact, while stress nuclear MPI is recommended for patients with intermediate or high pretest probability of CAD (22), it is not indicated for unselected patients with low pretest probability of CAD. This underscores the clinical need for a safe, accessible functional assessment tool in populations where stress testing is less routinely employed. Consequently, many symptomatic patients with non-obstructive CAD lack objective evidence of ischemia, which is precisely the prerequisite for considering invasive coronary function testing—the gold standard for endotype diagnosis. A tool like MCG, capable of detecting resting electrophysiological disturbances, could help identify those most likely to benefit from such definitive testing. However, it is crucial to acknowledge that the definitive diagnosis of underlying endotypes requires invasive coronary function testing, which was not performed in this study. Therefore, further validation of MCG's diagnostic performance against this reference standard is warranted before considering its broader clinical adoption. Specifically, prospective studies correlating quantitative MCG parameters with invasive coronary flow reserve (CFR) and microvascular resistance index (IMR) will be essential to define its diagnostic thresholds and clinical utility.

In this preliminary study, we focused on the non-invasive detection of ischemic patterns in patients with angina and non-obstructive coronary arteries using MCG. It is important to note that according to current EAPCI/COVADIS criteria, a definitive diagnosis of ANOCA endotypes requires invasive coronary function testing (CFR/IMR, acetylcholine provocation), which was not performed. Therefore, we describe these cases as “chest pain with non-obstructive CCTA and MCG findings suggestive of ischemia” rather than as definitively diagnosed ANOCA endotypes. Additionally, the reference standard for ischemia in this series was suboptimal, relying on either resting perfusion imaging or equivocal stress test results, which limits robust validation of the MCG abnormalities observed.

While promising, the clinical adoption of MCG faces practical hurdles. The system's high cost and limited availability outside specialized centers are initial barriers. Interpretation of MCG data requires specific expertise, indicating a learning curve and potential operator dependence; future development of automated analysis software is key to standardizing this process. Finally, defining its precise role within existing chest pain assessment pathways is necessary for effective integration. Addressing these challenges of cost, accessibility, and ease-of-use is essential for MCG to transition from a research tool to a routine clinical option for patients with suspected ANOCA.

Future studies that directly combine MCG with invasive functional testing or advanced non-invasive modalities (e.g., stress perfusion cardiac magnetic resonance) are warranted. Such work is essential to validate the role of MCG in endotype classification and to determine its diagnostic accuracy and potential clinical utility within the standardized ANOCA diagnostic pathway.

## Conclusion

The main finding of our study is that patients presenting with angina and found to have non-obstructive coronary arteries exhibit distinct MCG waveform characteristics, which provide a potential diagnostic basis for their acute ischemic presentations. These findings are clinically significant, as identification of underlying coronary microvascular dysfunction or coronary artery spasm in such patients may guide the initiation of appropriate pharmacological treatment and follow-up strategies. Our results suggest that MCG provides diagnostic information that is comparable to that obtained from nuclear stress testing. Consequently, it represents a simpler, less costly, radiation-free, and faster potential screening tool when clinically indicated. Notably, MCG may be particularly valuable as a screening tool, especially in patients with a low pretest probability of CAD. However, further studies with direct comparisons and formal assessment of sensitivity and specificity are needed to confirm its diagnostic performance relative to established imaging modalities.

## Data Availability

The original contributions presented in the study are included in the article/Supplementary Material, further inquiries can be directed to the corresponding authors.

## Appendix

Establishment of Normal Reference Ranges To define the normal reference ranges for MCG waveform parameters, we conducted a retrospective analysis of 1,011 adult subjects from a multicenter health screening database (Qilu Hospital of Shandong University, Linyi Hospital, and Guangdong Provincial Hospital of Chinese Medicine) collected between May 2021 and December 2024. Inclusion criteria included age 18-80 years and absence of self-reported cardiovascular disease, hypertension, or diabetes, as well as no related clinical symptoms. Exclusion criteria were applied for individuals with abnormal ECG findings, echocardiographic evidence of structural or functional heart disease, or prior cardiac interventions. All participants underwent standardized diagnostic evaluation to confirm eligibility. The final reference intervals were calculated as the 2.5th and 97.5th percentiles of the parameter distributions across the entire healthy cohort, ensuring robust representation of physiological variability. Waveform parameters were automatically analyzed, including T_amp, ratio_RT, and four amplitude points (k1amp–k4amp) obtained by equally dividing the ST segment from onset to offset into three segments.

**Appendix Table 1:** Normal reference ranges for MCG waveform parameters (central 95% interval).

	Definition	Feature	Reference
ST segment	Four amplitude points (k1–k4) obtained by equally dividing the ST segment from onset to offset into three segments.	k1amp	[0.114pT, 0.128pT]
		K2amp	[0.391pT, 0.421pT]
		K3amp	[0.745pT, 0.800pT],
		K4amp	[0.754pT, 0.819pT]
		ST-segment Morphology	Mild upward-sloping ST elevation, with no significant horizontal or downsloping ST depression.
T waveform	1) T amplitude: maximum magnetic field strength from isoelectric baseline to T-wave peak 2) R/T ratio: ratio of R-wave amplitude to T-wave amplitude	T amplitude	[2.858pT, 2.917pT]
		R/T ratio	[4.802, 4.891]
		T-Wave Morphology	In the R-wave positive channel, the T-wave is upright, without biphasic or inverted morphology.
